# New York College of Dental Surgery

**Published:** 1852-01

**Authors:** 


*New York College of Dental Surgery.*—We learn from the Announcement of the First Course of Lectures, recently received, that a college, under the above designation, has been instituted, at Syracuse, New York, with five professorships and one demonstratorship, and to which the following appointments have been made:

A. Westcott, M. D., D. D. S., Professor of the Theory and Practice of Dental Surgery and Dental Technology.

The chair of Institutes of Dentistry, Dental Hygiene, and Comparative Dental Anatomy, has not yet been filled.

A. B. Shipman, M. D., Professor of Anatomy and Physiology, and General Principles of Surgery.

Thomas Spencer, M. D, Professor of Special Pathology and Therapeutics.

R. F. Stevens, M. D., Professor of Chemistry.

Daniel Van Denburgh, D. D. S., Demonstrator of Mechanical and Surgical Dentistry.

The first course of lectures commenced on the first Monday in December, 1851, and will continue fifteen weeks. The duties of the vacant chair, will, we understand, be performed, until it is filled, by professor Westcott. With regard to the number in the class of the present session we are ignorant, though we were informed by a friend that it was expected there would be about thirty in attendance. We hope the friends of the school may realize their most ardent anticipations in the enterprize.

